# Different adiposity indices and their association with blood pressure and hypertension in middle-aged urban black South African men and women: findings from the AWI-GEN South African Soweto Site

**DOI:** 10.1186/s12889-018-5443-4

**Published:** 2018-04-19

**Authors:** Pedro T. Pisa, Lisa K. Micklesfield, Juliana Kagura, Michele Ramsay, Nigel J. Crowther, Shane A. Norris

**Affiliations:** 1 MRC/Wits Developmental Pathways for Health Research Unit, Department of Paediatrics, Faculty of Health Sciences, University of the Witwatersrand, Johannesburg, South Africa; 2 Sydney Brenner Institute for Molecular Bioscience and Division of Human Genetics, University of the Witwatersrand, Johannesburg, South Africa; 3 Department of Chemical Pathology, National Health Laboratory Service, University of the Witwatersrand Medical School, Johannesburg, South Africa

**Keywords:** Body composition, Anthropometry, Blood pressure, Hypertension, South Africa, Adults, AWI GEN

## Abstract

**Background:**

To report associations between different adiposity indices [anthropometric and dual-energy X-ray absorptiometry (DXA) measures] and blood pressure (BP) and hypertension in urban black South African adults.

**Methods:**

Anthropometric and DXA whole body measures were performed on 1026 men and 982 women. Participants were classified as being hypertensive if they had a systolic BP (SBP) ≥ 140 mmHg and/or diastolic (DBP) ≥ 90 mmHg. Within each gender the relationship of adiposity with BP and hypertension risk was assessed using linear and logistic regression models respectively. Bivariate models were computed for each body composition variable. Furthermore, we computed a multiple regression model to illustrates how body composition parameters are associated with the outcome variables independent of each other.

**Results:**

The males were significantly taller and had a higher fat free soft tissue mass (FFSTM), DBP and socio-economic status, and were more likely to use tobacco and be hypertensive (48.0% vs. 38.8%). The females had higher body mass index (BMI), waist circumference (WC), fat mass (FM), subcutaneous adipose tissue (SAT), visceral adipose tissue (VAT), FM/FFSTM ratio and body fat % than males. All body composition parameters were positively associated with hypertension. In both males and females, the FM/FFSTM ratio associated the strongest with hypertension illustrating the following odds ratios [males: 70.37 (18.47, 268.16) *p* ≤ 0.001; females 2.48 (0.86,7.21) *p* = 0.09]. The multiple regression model, indicated that the VAT and WC significantly associated with both SBP and DBP in the men and women respectively, whilst WC was the only significant predictor for hypertension.

**Conclusions:**

All body composition parameters were associated with hypertension and FM/FFSTM ratio showed the strongest relationship. It was reassuring that WC remains a useful measure of central adiposity that can be used as a risk indicator for hypertension if more sophisticated measures are not available. Furthermore, our data in part, implies that reducing abdominal adiposity in aging adults could contribute to reducing the risk of elevated blood pressure and hypertension.

**Electronic supplementary material:**

The online version of this article (10.1186/s12889-018-5443-4) contains supplementary material, which is available to authorized users.

## Background

The Global Burden of Diseases, Injuries, and Risk Factors study reported that the largest contributor to global disability-adjusted life-years (DALYs) was high systolic blood pressure [1]. Additionally, in a pooled analysis of 1479 studies that had measured the blood pressures of 19.1 million adults globally, data indicated Sub-Saharan Africa as one of the regions that had the highest blood pressure levels [2]. The number of adults with raised blood pressure increased from 594 million in 1975 to 1.13 billion in 2015 worldwide, with the increase largely in low-income and middle-income countries (LMICs) [2].

The epidemiological health transition, which is attributed to rapid urbanisation, has been pivotal in partly explaining the emergence of non-communicable diseases (NCDs) in many LMICs including South Africa [3–5]. Data from the recent national general household survey conducted by Statistics South Africa, reported a 10.4% prevalence of self-reported hypertension diagnosed by a medical professional [6]. The observed prevalence increased with age in both genders and was higher in women in all age groups [6]. In the same survey, the age standardised incidence rates (ASR) were highest among black African women, but lowest in black African men [6].

Obesity, a known independent risk factor for NCDs, has become a major public health concern in South Africa. In part, this has largely been associated with various factors including shifts from prudent dietary patterns, to adoption of westernised diets, and diminished physical activity [3, 5, 7]. Urban communities have been reported to be at higher risk for obesity and its associated diseases including hypertension [3, 8, 9]. Many studies have demonstrated a positive association between obesity and blood pressure in adult populations using both longitudinal and cross sectional data [10–12]. Data from a recent longitudinal analysis that only included urban black South African women residing in Soweto, reported a 57.1% cumulative incidence of hypertension [13]. Furthermore, baseline BP was the most significant predictor of hypertension, and central adiposity was associated with greater odds of developing hypertension than total adiposity [13]. However, little is known regarding the association between body composition and blood pressure in black African men.

Body mass index (BMI) a measure of overall body size has been reported to be a useful but crude indicator of body composition and adiposity, as it cannot adequately distinguish between whole body fat and lean mass [14], and does not take into account body fat distribution. Furthermore, not much data is available on how DXA-derived measures, which may provide more objective parameters of body composition associate with hypertension in Africans undergoing transition. The aim of this study was to examine the associations between anthropometric and DXA-derived body composition parameters, and blood pressure and hypertension, among urban black South African men and women.

## Methods

### Design and study participants

Data from this cross-sectional study was collected between 2011 and 2015 on black South African men (*n* = 1026) and women (*n* = 982) residing in Soweto, Johannesburg. The females included are also the caregivers of the Birth to Twenty Plus Cohort study, the largest longitudinal birth cohort on childhood development and health in Africa to date. The men were part of a larger African genomics study referred to as AWI-GEN (Africa Wits-INDEPTH partnership for Genomic Research). Though separate studies, both men and women consented to be part of this current study, and participants had to have complete data on both the exposure and outcome variables of interest as needed by the current study. All data were collected at the same research institute and time-period and the same instruments, tools and protocols were used.

### Outcome measurements

#### Blood pressure measurements

SBP and DBP were measured in triplicate in a seated position using an Omron machine (Kyoto, Japan) on the right arm using an appropriate-sized cuff, with a 2-min interval between the measurements. The average of the second and third measurements was used for analyses. Hypertension status was defined as SBP ≥ 140 mmHg and/or DBP ≥90 mmHg (stage 2) or being on hypertension medication.

### Exposure variables

#### Anthropometry

Weight and height were measured by a trained research assistant using a calibrated electronic scale and stadiometer, respectively, on participants wearing light clothes and barefoot. The BMI was computed from the weight and height measures (weight in kg/height in metres^2^). Waist circumference (WC) was measured with a soft measuring tape at the level of the umbilicus.

#### Body composition measures using dual-energy X-ray absorptiometry (DXA)

The DXA-derived body composition parameters were measured using a Hologic Discovery A (S/N 83145) DXA machine. Participants removed clothing and all metal objects, and wore surgical gowns for these measurements. Using software version 12.5.7 (Bedford, MA, USA) whole body scans were completed to determine sub-total (whole body minus head) fat mass (FM) and fat free soft tissue mass (FFSTM). Visceral adipose tissue (VAT) and subcutaneous adipose tissue (SAT) were measured using DXA which has been shown to perform well as a clinical read of VAT from a computerized tomography scan [15]. During data collection, a phantom was scanned each morning to determine the coefficient of variance (CV) of the DXA machine and the coefficient of variation was less than 0.5% for all parameters.

#### Covariate measurements

Household SES was estimated using a count of the major household amenities, namely a motor vehicle, refrigerator, washing machine, DSTV/satellite, microwave and possessing a cell phone. Tobacco use was evaluated by asking the participant if they had ever been exposed to tobacco through smoking, chewing or taking snuff. A voluntary HIV antibody test, Alere Determine™ HIV-1/2 (Alere San Diego, Inc. San Diego, CA) was offered to all participants and the process included pre- and post-test counselling sessions. Those who reported that they were HIV positive were not re-tested. If positive, participants were referred to local HIV clinics for a confirmatory serological test, CD4 count and further care.

#### Statistical analysis

Sample characteristics were compared between men and women using an independent t-test and Pearson chi-squared test for continuous and categorical variables, respectively. All analyses are presented by sex. Bivariate linear regressions were computed to assess the association of various body composition parameters namely BMI, WC, FM, FFSTM, FM/FFSTM ratio, VAT and SAT, with SBP, DBP and hypertension. Regression coefficients and odd ratios (and their 95% CIs) are presented. Furthermore, we computed multiple regression models to explore how body composition parameters are associated with the outcome variables independent of the other body composition parameters. All the variables included in the paper (SES, age, education, HIV status, smoking plus all the anthropometric variables) are scientifically plausible determinants of blood pressure. Supplementary analysis was computed, which included these plausible determinants in each of the multivariable regression models for SBP, DBP and hypertension in both sexes. Variance inflation factors (VIFs) were calculated to understand the level of collinearity for each variable in the regression models, this was followed by removing the variables with high VIFs (using a cut-off of 5) and the final models are presented after performing backward stepwise removal of non-significant variables (SES, age, education, and smoking were removed for SBP, DBP and hypertension by this process). The variables that were removed due to collinearity for SBP and DBP for males were whole body fat mass, BMI, WC and SAT, whereas whole body total fat, BMI, SAT and whole body total FFSTM were removed for SBP and DBP for females. As for hypertension, in both males and females whole body fat, whole body FFSTM, SAT and BMI were removed. Pearson correlations were computed for all anthropometric variables and showed that body fat % correlates very strongly with all other anthropometric variables in both males and females, but more importantly with whole body fat mass and the ratio, FM: FFSTM. Therefore, it was not included any of the regression models. The Statistical Package for Social Sciences (SPSS) version 20 was used for analysis. We performed a two-sided statistical test at a significance level *P* value < 0.05.

## Results

Table 1 compares the anthropometric, DXA-derived body composition, blood pressure, lifestyle and socio-demographic parameters by sex. No difference in age was observed between the males and females however the males were significantly taller and had a higher FFSTM, DBP, SES score, and were more likely to use tobacco and be hypertensive. The females were more likely to have a secondary education, were heavier, and had significantly higher body fat measures including BMI, WC, FM, SAT, VAT, FM/FFSTM ratio and body % fat.

**Table 1:** Comparison of socio-demographic, blood pressure, dual-energy X-ray (DXA) derived body composition, anthropometric and lifestyle factors between males and females

Variable	Males (*n* = 1026)	Females (*n* = 982)	
	Mean (SD) /%	Mean (SD)/%	*P*-value
Demographics			
Age (years)	49.42 (6.03)	49.16 (6.08)	0.314
Proportion with secondary education (%)	72.9%	78.6%	0.003
SES asset score	4.49 (1.45)	3.02 (1.53)	≤0.001
Outcomes: Blood pressure			
Systolic blood pressure (mmHg)	131.88 (21.03)	132.49 (21.51)	0.521
Diastolic blood pressure (mmHg)	89.31 (13.18)	81.78 (9.45)	≤0.001
Hypertensive (%)	48.0%	38.8%	≤0.001
DXA derived body composition and anthropometric parameters			
Height (m)	1.71 (0.06)	1.58(0.06)	≤0.001
Weight (kg)	73.13 (17.52)	82.65(18.20)	≤0.001
Body mass index (kg/m^2^)	24.96 (5.65)	33.10(6.97)	≤0.001
Waist circumference (cm)	89.22 (14.81)	98.46(14.45)	≤0.001
Whole body fat mass (FM) (kg)	18.74 (83.70)	32.97 (10.10)	≤0.001
Whole body fat free soft tissue mass (FFSTM) (kg)	51.32 (85.32)	44.96 (70.81)	≤0.001
FM/FFSTM	0.36 (0.12)	0.73 (0.16)	≤0.001
Body fat %	24.71 (6.30)	40.32 (5.78)	≤0.001
Subcutaneous adipose tissue (cm^2^)	190.06 (125.40)	454.67 (153.60)	≤0.001
Visceral adipose tissue (cm^2^)	84.56 (43.62)	99.83 (40.42)	≤0.001
Lifestyle factors			
HIV negative (%)	80.4%	79.4%	0.668
Tobacco users (%)	69.8%	28.5%	≤0.001

Table 2 presents bivariate regression models between body composition parameters, with SBP and DBP by sex. All body composition parameters were positively associated with SBP in both men and women. All associations had higher beta coefficients for SBP in men than women. Bivariate models indicate that the FM/FFSTM ratio followed by BMI to have the strongest association with SBP in both sexes.

**Table 2:** Bivariate regression models examining the association between body composition parameters and systolic/diastolic blood pressure in urban male and female black South African adults

	Male		Female	
	B (95% CI)	*p* value	B (95% CI)	*p* value
Systolic blood pressure (mmHg)				
Body mass index (kg/m^2^)	0.91 (0.69;1.14)	≤0.001	0.29 (0.10;0.49)	0.003
Waist circumference (cm)	0.38 (0.30;0.46)	≤0.001	0.22 (0.13;0.31)	≤0.001
hole body fat mass (FM) (kg)	0.61 (0.40;0.70)	≤0.001	0.20 (0.03;0.40)	0.02
Whole body fat free soft tissue mass (FFSTM) (kg)	0.44 (0.27;0.62)	≤0.001	0.28 (0.04;0.53)	0.02
FM/FFSTM	42.93 (30.26;55.60)	≤0.001	10.61 (−0.10; 21.32)	0.05
Body fat %	0.85 (0.61;1.10)	≤0.001	0.35 (0.05;0.65)	0.02
Subcutaneous adipose tissue (cm^2^)	0.04 (0.03;0.05)	≤0.001	0.02 (0.001;0.03)	0.001
Visceral adipose tissue (cm^2^)	0.15 (0.11;0.18)	≤0.001	0.07 (0.03;0.11)	0.001
Diastolic blood pressure (mmHg)				
Body mass index (kg/m^2^)	0.60 (0.46;0.74)	≤0.001	0.28 (0.20;0.36)	≤0.001
Waist Circumference (cm)	0.25 (0.20;0.30)	≤0.001	0.17 (0.13;0.21)	≤0.001
Whole body fat mass (FM) (kg)	0.39 (0.27;0.50)	≤0.001	0.18 (0.10;0.26)	≤0.001
Whole body fat free soft tissue mass (FFSTM) (kg)	0.29 (0.18;0.40)	≤0.001	0.21 (0.10;0.32)	≤0.001
FM/FFSTM	26.72 (18.80;34.63)	≤0.001	9.62 (4.72;14.52)	≤0.001
Body fat %	0.53 (0.38;0.68)	≤0.001	0.28 (0.14;0.42)	≤0.001
Subcutaneous adipose tissue (cm^2^)	0.03 (0.02;0.03)	≤0.001	0.01 (0.007;0.02)	≤0.001
Visceral adipose tissue (cm^2^)	0.09 (0.07;0.11)	≤0.001	0.05 (0.03;0.07)	≤0.001

As for DBP, positive associations were observed with all body composition parameters in both men and women. Similar to SBP all regression coefficients were higher in men than women. The greatest coefficient on DBP observed is with FM/FFSTM ratio, followed BMI and body fat % in both sexes (Table 2).

Table 3 presents bivariate logistic regression models examining the associations between body composition parameters and hypertension by sex. All body composition parameters were positively associated with hypertension in men, with the highest odds ratio being for the FM/FFSTM ratio, followed by BMI. In the women, all body composition parameters except FM/FFSTM ratio and body fat % were significantly associated with hypertension (*p* ≤ 0.001).

**Table 3:** Bivariate logistic regression models presenting different body composition parameters as predictors of hypertension in urban black male and female South African adults

	Male		Female	
	Odds Ratio (95% CI)	*p* value	Odds Ratio (95% CI)	*p* value
Body mass index (kg/m^2^)	1.11 (1.08;1.14)	≤0.001	1.03 (1.01;1.05)	0.003
Waist Circumference (cm)	1.04 (1.03;1.05)	≤0.001	1.02 (1.01;1.03)	≤0.001
Whole body fat mass (FM) (kg)	1.06 (1.04;1.08)	≤0.001	1.02 (1.00;1.04)	0.04
Whole body fat free soft tissue mass (FFSTM) (kg)	1.04 (1.03;1.06)	≤0.001	1.03 (1.00;1.05)	0.03
FM/FFSTM	70.37 (18.47; 268.16)	≤0.001	2.48 (0.86;7.21)	0.09
Body fat %	1.09 (1.06; 1.12)	≤0.001	1.03 (1.00;1.06)	0.06
Subcutaneous adipose tissue (cm^2^)	1.004 (1.003;1.005)	≤0.001	1.001 (1.000;1.002)	0.03
Visceral adipose tissue (cm^2^)	1.01 (1.00;1.02)	≤0.001	1.007 (1.003;1.01)	0.001

However, after computing multiple regression models to explore how body composition parameters are associated with the outcome variables independent of each other (Additional file 1: Table S1). We observed that the VAT and WC were only parameters significantly associated with both SBP and DBP in the men and women, respectively. As for hypertension, in both men and women, WC was the only significant predictor.

## Discussion

In the present study we examined associations between various adiposity indicators and blood pressure and odds of being hypertensive among urban black middle-aged South African adults. A key finding is that central adiposity in both men and women associates with elevated blood pressure and greater likelihood of being hypertensive.

This study confirms the contribution of body composition to blood pressure and hypertension risk as shown by others [13, 16–19], but takes it one step further in trying to understand the relationship between the different body composition including DXA derived components and how they associate with BP and hypertension independently and after adjusting for specific variables. To contextualize our findings from the bivariate models, for one-unit increase in the FM/FFSTM ratio, there is a 32 times greater risk for being hypertensive in the men and 2.3 times for the women. Using BMI, for every shift in BMI category (5 BMI units; normal (20 kg/m^2^) to overweight (25 kg/m^2^) or overweight to obese (30 kg/m^2^)) there is 55% greater odds for hypertension for men and 15% for women. Using WC, with an increase of WC by 5 cm, there is 20% greater odds for being hypertensive for males and 10% for females. The data suggests that the risk of elevated blood pressure or hypertension due to higher adiposity is much greater in men than women. However, the multiple regression models, confirms that WC in both men and women is significantly associated with hypertension after adjusting for other body composition parameters and by removing the variables with high VIFs to account for collinearity despite the limitations associated with this approach.

### Overall adiposity (BMI and body fat %)

Data from the US National Health and Nutrition Examination Surveys (NHANES) indicates a positive association between BMI and hypertension, reporting a prevalence of 45.2%, 27.8% and 15.3% for those with a BMI ≥ 30 kg/m^2^, 25–29.9 kg/m^2^ and < 25 kg/m^2^ respectively in adult men and women (Wang and Wang, 2004). Data from a cohort study including 4 sub-Saharan countries indicated that BMI was one of the primary factors found to be associated with hypertension [20]. A longitudinal study conducted among young white and black adults showed that weight gain and not baseline weight was more important for significant increase in blood pressure [21]. Furthermore, the Framingham Heart study reported that, in comparison to normal weight individuals, adjusted relative risks for developing hypertension were higher in obese individuals in both genders [12]. In contrast to our findings, the “Framingham Heart Study” reported higher relative risks for being hypertensive in women compared to men [12]. This could in part be explained by the fact that for the same body size, differences in body fat distribution between black and white South African women have been reported [22, 23], with the latter group having more visceral fat than the former. Other than the current study, no data are available that compare body fat distribution between African men and women in relation to blood pressure and hypertensive risk.

### Central adiposity (WC and VAT)

Waist circumference, a well-accepted indicator for central body fat, has been reported to be the strongest independent predictor of SBP and DBP compared to other cardio-metabolic risk factors including fasting insulin levels and resistance in both men and women [19, 24]. Our findings show that WC was the only measure in both men and women that was significantly associated with increased odds of being hypertensive in the multiple regression analysis. In another South African study, WC and VAT were significant determinants of all metabolic risk variables including blood pressure, in both white and black South African women [25]. The mechanisms by which central adiposity may cause hypertension are not fully understood. However, there are a number of hypotheses which include insulin resistance, secretion of angiotensinogen and inflammatory cytokines by adipocytes, accumulation of VAT that disturbs the renal medulla and consequently increases sodium reabsorption [17].

### FM/FFSTM ratio and gender differences

FM/FFSTM ratio seemed to be the strongest predictor of hypertension in the bivariate analysis. This could be attributed to this variable being a ratio and it additionally strongly correlates to other BC parameters. Studies have explored different body composition ratios and how they predict various CVD risk factors including BP. Findings from the Korean National Health Nutrition Examination Survey (KNHANES) also found that sarcopenic obese subjects had greater risk of hypertension compared to only obese or sarcopenia [26].

Significant sex differences in blood pressure and hypertension have been observed in other studies [27] where men had higher BP and were more likely to be hypertensive compared to women at similar ages. Blood pressure has been shown to increase with aging in both genders [27], though comparatively higher levels remain in men [27]. However, the Cardiovascular Risk in Black South Africans study conducted in the urban black population of Cape Town reported an age-standardised hypertension prevalence of 38.9% with similar rates observed between men and women [18]. Women have been suggested to be less responsive to the epidemiological health transition associated with urbanisation, thus suggesting a buffering effect for women against environmental factors related to increased blood pressure [9]. However, the mechanisms responsible for hypertension in the current population could be complicated by co-morbid conditions, which may be different between the sexes, for example stress, depression, and other factors including diet (salt intake) and physical activity patterns that were not controlled for in the current study.

### HIV and blood pressure

In males, systolic blood pressure was lower in HIV-positive compared to HIV-negative participants and there was an observed directional risk of hypertension to be lower in the former group (*p* = 0.06). No such effects were observed in females. A meta-analysis of studies conducted in Africa on the effect of HIV infection and therapy on cardio-metabolic variables showed that, after adjustment for possible confounding variables, systolic but not diastolic blood pressure was lower in HIV-positive participants [28]. Furthermore, a study conducted in South Africa showed that HIV infection was negatively associated with an increase in blood pressure over a 5-year period [29]. The reason for the negative association observed between blood pressure and HIV infection in these studies is not fully understood, and clearly requires further investigation. However, in this study, data on HIV therapy and medication was not available, and only HIV status was used.

The major strengths of this study are that we included multiple measures of adiposity, and had a large sample size for both sexes, whilst adjusting for a number of known covariates. In the current analysis, elimination and removal of other independent variables from the model was primarily based on a threshold set for VIFs (and based on theoretical motivation as all included independent variables are known to be associated to the outcomes for the current study). This however means that the models have limitations in that possible biases, and errors in interpreting associations are possible. By eliminating one or two independent variables, that are highly correlated with the other, this might respecify the model. This approach allows for the model to shift and this often mean that the theory being tested by the model also changes hence leading to a model that is not theoretically well motivated. However, this remains a useful method to resolving collinearity in regression models. Not all data known to influence blood pressure were available to be adjusted for in the analysis. Additionally, this study was cross-sectional and thus causality cannot be inferred.

## Conclusions

All body composition parameters were associated with hypertension and FM/FFSTM ratio showed the strongest relationship. It was reassuring that WC remains a useful measure of central adiposity that can be used as a risk indicator for hypertension if more sophisticated measures are not available. Furthermore, our data in part, implies that reducing abdominal adiposity in aging adults could contribute to reducing the risk of elevated blood pressure and hypertension.

## Additional file

Additional file 1: **Table S1.** Multivariate regression models examining the association between body composition parameters and blood pressure and hypertension in urban male and female black South African*. (DOCX 13 kb)

## Abbreviations

ASR: Age standardised incidence rates
AWI-GEN: Africa Wits-INDEPTH partnership for Genomic Research
BP: Blood pressure
BMI: Body mass index
CV: Coefficient of variance
DBP: Diastolic blood pressure
DALYs: Disability-adjusted life-years
DXA: Dual-energy X-ray absorptiometry
FFSTM: Fat free soft tissue mass
FM: Fat mass
HIV: Human immune-deficiency virus
KNHANES: Korean National Health Nutrition Examination Survey
LMICs: Low-income and middle-income countries
NHANES: National Health and Nutrition Examination Surveys
NCDs: Non-communicable diseases
SES: Socio economic status
SPSS: Statistical Package for Social Sciences
SAT: Subcutaneous adipose tissue
SBP: Systolic blood pressure
VIFs: Variance inflation factors
VAT: Visceral adipose tissue
WC: Waist circumference
