# Supplementary material for: Different adiposity indices and their association with blood pressure and hypertension in middle-aged urban black South African men and women: findings from the AWI-GEN South African Soweto Site
Source: BMC Public Health. 2018 Apr 19;18:524. doi: 10.1186/s12889-018-5443-4 (PMC5907712; doi:10.1186/s12889-018-5443-4)
Supplement: Supplementary file 1 — Table S1. Multivariate regression models examining the association between body composition parameters and blood pressure and hypertension in urban male and female black South African*. (DOCX 13 kb) [file 12889_2018_5443_MOESM1_ESM.docx]

| **Supplementary Table 1** Multivariate regression models examining the association between body composition parameters and blood pressure and hypertension in urban male and female black South African* | | |
| --- | --- | --- |
| Male | | |
| **Systolic blood pressure (mmHg)** | **B (95% CI)** | **p value** |
| Visceral adipose tissue (cm^2^) | 0.12 (0.08;0.16) | ≤0.001 |
| Age (years) | 0.55 (0.30;0.81) | ≤0.001 |
| HIV | -4.46 (-8.42;-0.51) | 0.03 |
| **Diastolic blood pressure (mmHg)** | **B (95% CI)** | **p value** |
| Visceral adipose tissue (cm^2^) | 0.09 (0.06;0.11) | ≤0.001 |
| **Hypertension** | **Odds Ratio (95% CI)** | **p value** |
| Age | 1.03 (1.00; 1.05) | 0.02 |
| HIV | 0.72 (0.50; 1.02) | 0.06 |
| Waist Circumference (cm) | 1.04 (1.03;1.05) | ≤0.001 |
| Female | | |
| **Systolic blood pressure (mmHg)** | **B (95% CI)** | **p value** |
| Age (years) | 0.92 (0.69;1.15) | ≤0.001 |
| Waist Circumference (cm) | 0.18 (0.09;0.27) | ≤0.001 |
| **Diastolic blood pressure (mmHg)** | **B (95% CI)** | **p value** |
| Waist Circumference (cm) | 0.17 (0.13;0.21) | ≤0.001 |
| **Hypertension** | **Odds Ratio (95% CI)** | **p value** |
| Age (years) | 1.07 (1.04;1.09) | ≤0.001 |
| Waist Circumference (cm) | 1.02 (1.01;1.03) | ≤0.001 |

* Significant beta and Odd ratios in the final multivariate models (after removing the variables with VIFs ≥ 5). Followed by performing backward stepwise removal of non-significant variables. The variables that were removed due to collinearity for SBP and DBP for males were whole body fat mass, BMI, WC and SAT. Whereas whole body total fat, BMI, SAT and whole body total FFSTM were removed for SBP and DBP for females. As for hypertension, in both males and females whole body fat, whole body FFSTM, SAT and BMI were removed
